# (2*E*)-2-(Thio­phen-2-yl­methyl­idene)-1,2,3,4-tetra­hydro­naphthalen-1-one

**DOI:** 10.1107/S1600536812029169

**Published:** 2012-06-30

**Authors:** Abdullah M. Asiri, Hassan M. Faidallah, Khalid A. Alamry, Seik Weng Ng, Edward R. T. Tiekink

**Affiliations:** a Center of Excellence for Advanced Materials Research (CEAMR), King Abdulaziz University, PO Box 80203, Jeddah 21589, Saudi Arabia; b Chemistry Department, Faculty of Science, King Abdulaziz University, PO Box 80203, Jeddah 21589, Saudi Arabia; c Department of Chemistry, University of Malaya, 50603 Kuala Lumpur, Malaysia

## Abstract

In the title compound, C_15_H_12_OS, the cyclo­hexene ring has a twisted boat conformation with the C atom between the ketone and methyl­ene atom and this methyl­ene C atom lying 0.280 (3) and 0.760 (3) Å, respectively, from the plane through the remaining four atoms (r.m.s. deviation = 0.004 Å). The dihedral angle between the benzene and thio­phene rings [21.64 (9)°] indicates an overall twist in the mol­ecule. The thio­phene S and ketone O atoms are *anti*, an orientation that allows the close approach of these atoms [3.3116 (17) Å] in the crystal structure and which leads to the formation of helical supra­molecular chains along the *c* axis.

## Related literature

For the activity of related species developed for the treatment of Chagas disease, see: Vera-DiVaio *et al.* (2009 ▶). For a related structure, see: Asiri *et al.* (2012 ▶).
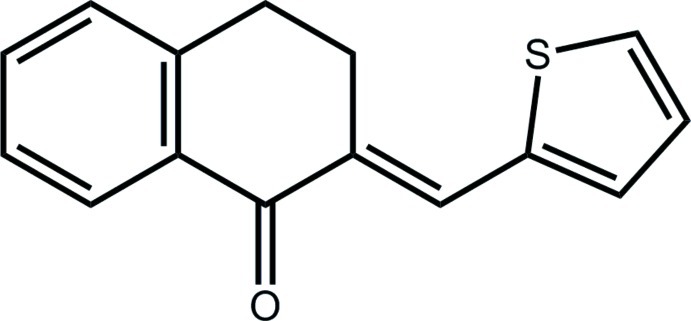


## Experimental

### 

#### Crystal data

C_15_H_12_OS
*M* *_r_* = 240.31
Orthorhombic, 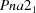

*a* = 24.7989 (10) Å
*b* = 3.9976 (2) Å
*c* = 11.3163 (5) Å
*V* = 1121.85 (9) Å^3^
*Z* = 4
Mo *K*α radiation
μ = 0.27 mm^−1^
*T* = 100 K
0.35 × 0.30 × 0.25 mm

#### Data collection

Agilent SuperNova Dual diffractometer with an Atlas detector
Absorption correction: multi-scan (*CrysAlis PRO*; Agilent, 2012 ▶) *T*_min_ = 0.812, *T*_max_ = 1.000
7054 measured reflections
2528 independent reflections
2383 reflections with *I* > 2σ(*I*)
*R*_int_ = 0.029

#### Refinement

*R*[*F* ^2^ > 2σ(*F* ^2^)] = 0.037
*wR*(*F* ^2^) = 0.097
*S* = 1.03
2528 reflections
154 parameters
1 restraint
H-atom parameters constrained
Δρ_max_ = 0.32 e Å^−3^
Δρ_min_ = −0.25 e Å^−3^
Absolute structure: Flack (1983 ▶), 1171 Friedel pairs
Flack parameter: 0.07 (10)

### 

Data collection: *CrysAlis PRO* (Agilent, 2012 ▶); cell refinement: *CrysAlis PRO*; data reduction: *CrysAlis PRO*; program(s) used to solve structure: *SHELXS97* (Sheldrick, 2008 ▶); program(s) used to refine structure: *SHELXL97* (Sheldrick, 2008 ▶); molecular graphics: *ORTEP-3 for Windows* (Farrugia, 1997 ▶) and *DIAMOND* (Brandenburg, 2006 ▶); software used to prepare material for publication: *publCIF* (Westrip, 2010 ▶).

## Supplementary Material

Crystal structure: contains datablock(s) global, I. DOI: 10.1107/S1600536812029169/gg2087sup1.cif

Structure factors: contains datablock(s) I. DOI: 10.1107/S1600536812029169/gg2087Isup2.hkl

Supplementary material file. DOI: 10.1107/S1600536812029169/gg2087Isup3.cml

Additional supplementary materials:  crystallographic information; 3D view; checkCIF report

## Figures and Tables

**Fig. 1.**
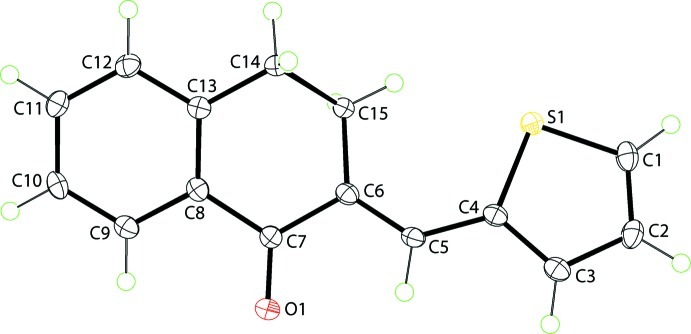
The molecular structure of (I) showing the atom-labelling scheme and displacement ellipsoids at the 50% probability level.

**Fig. 2.**
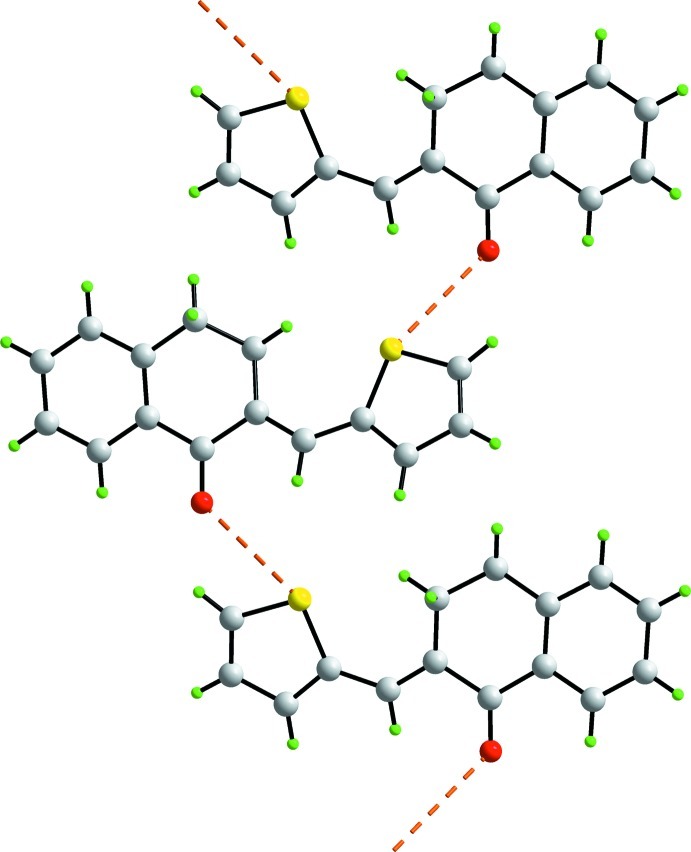
A view of the helical supramolecular chain along the c axis in (I) mediated by S···O interactions shown as orange dashed lines.

## supplementary crystallographic information

### Comment

In continuation of structural studies on tetrahydronaphthalen-1-one derivatives (Asiri *et al.*, 2012), the crystal and molecular structure of the title compound, 2-thiophen-2-ylmethylene-3,4-dihydro-2*H*-naphthalen-1-one (I), was investigated. Interest in this class of compound stems from their putative activity against Chagas disease (Vera-DiVaio *et al.*, 2009).

In (I), Fig. 1, the cyclohexene ring has a twisted boat conformation with the C6 and C15 atoms lying, respectively, 0.280 (3) and 0.760 (3) Å from the plane through the remaining four atoms which have a r.m.s. deviation = 0.004 Å. Overall, the molecule is twisted with the dihedral angle between the benzene and thiophen-2-yl rings being 21.64 (9)°. The conformation about the exocyclic methylidene C5═C6 [1.349 (3) Å] is *E*. The thiophen-2-yl-S and ketone-O atoms are *anti*.

In the crystal packing, weak π—π interactions are noted between translationally related benzene rings, *i.e*. inter-centroid distance = 3.9976 (11) Å (symmetry operation *x*, 1 + *y*, *z)* which lead to stacks along the *b* axis. Other than these, the most prominent interactions appear to be of the type S···O, *i.e*. S1···O1^i^ = 3.3116 (17) Å for *i*: 1 - *x*, 1 - *y*, 1/2 + *z*. The result is the formation of helical supramolecular chains along the *c* axis, Fig. 2.

### Experimental

A solution of the 2-thiophen-2-carboxaldehyde (1.1 g, 0.01 *M*) in ethanol (20 ml) was added to a stirred solution of 1-tetralone (1.46 g,0.0 1*M*) in ethanolic KOH (20 ml, 20%). Stirring was maintained at room temperature for 6 h. The reaction mixture was then poured onto water (200 ml) and set aside overnight. The precipitated solid product was collected by filtration, washed with water, dried and recrystallized from its ethanol solution. *M*.pt: 351–352 K. Yield: 92%.

### Refinement

Carbon-bound H-atoms were placed in calculated positions [C—H = 0.95–0.99 Å, *U*_iso_(H) = 1.2*U*_eq_(C)] and were included in the refinement in the riding model approximation. Owing to poor agreement, one reflection, *i.e*. (6 3 - 3), was omitted from the final refinement.

### Crystal data

**Table:**

C_15_H_12_OS	*D*_x_ = 1.423 Mg m^−^^3^
*M**_r_* = 240.31	Melting point: 351 K
Orthorhombic, *P**n**a*2_1_	Mo *K*α radiation, λ = 0.71073 Å
Hall symbol: P 2c -2n	Cell parameters from 3882 reflections
*a* = 24.7989 (10) Å	θ = 2.4–27.5°
*b* = 3.9976 (2) Å	µ = 0.27 mm^−^^1^
*c* = 11.3163 (5) Å	*T* = 100 K
*V* = 1121.85 (9) Å^3^	Prism, light-brown
*Z* = 4	0.35 × 0.30 × 0.25 mm
*F*(000) = 504	

### Data collection

**Table:**

Agilent SuperNova Dual diffractometer with an Atlas detector	2528 independent reflections
Radiation source: SuperNova (Mo) X-ray Source	2383 reflections with *I* > 2σ(*I*)
Mirror monochromator	*R*_int_ = 0.029
Detector resolution: 10.4041 pixels mm^-1^	θ_max_ = 27.6°, θ_min_ = 2.4°
ω scan	*h* = −32→23
Absorption correction: multi-scan (*CrysAlis PRO*; Agilent, 2012)	*k* = −5→4
*T*_min_ = 0.812, *T*_max_ = 1.000	*l* = −14→14
7054 measured reflections	

### Refinement

**Table:**

Refinement on *F*^2^	Secondary atom site location: difference Fourier map
Least-squares matrix: full	Hydrogen site location: inferred from neighbouring sites
*R*[*F*^2^ > 2σ(*F*^2^)] = 0.037	H-atom parameters constrained
*wR*(*F*^2^) = 0.097	*w* = 1/[σ^2^(*F*_o_^2^) + (0.0596*P*)^2^ + 0.206*P*] where *P* = (*F*_o_^2^ + 2*F*_c_^2^)/3
*S* = 1.03	(Δ/σ)_max_ = 0.001
2528 reflections	Δρ_max_ = 0.32 e Å^−^^3^
154 parameters	Δρ_min_ = −0.25 e Å^−^^3^
1 restraint	Absolute structure: Flack (1983), 1171 Friedel pairs
Primary atom site location: structure-invariant direct methods	Flack parameter: 0.07 (10)

### Special details

**Table:**

Geometry. All e.s.d.'s (except the e.s.d. in the dihedral angle between two l.s. planes) are estimated using the full covariance matrix. The cell e.s.d.'s are taken into account individually in the estimation of e.s.d.'s in distances, angles and torsion angles; correlations between e.s.d.'s in cell parameters are only used when they are defined by crystal symmetry. An approximate (isotropic) treatment of cell e.s.d.'s is used for estimating e.s.d.'s involving l.s. planes.
Refinement. Refinement of *F*^2^ against ALL reflections. The weighted *R*-factor *wR* and goodness of fit *S* are based on *F*^2^, conventional *R*-factors *R* are based on *F*, with *F* set to zero for negative *F*^2^. The threshold expression of *F*^2^ > σ(*F*^2^) is used only for calculating *R*-factors(gt) *etc*. and is not relevant to the choice of reflections for refinement. *R*-factors based on *F*^2^ are statistically about twice as large as those based on *F*, and *R*- factors based on ALL data will be even larger.

### Fractional atomic coordinates and isotropic or equivalent isotropic displacement parameters (Å^2^)

**Table:**

	*x*	*y*	*z*	*U*_iso_*/*U*_eq_	
S1	0.538955 (17)	0.26862 (11)	0.50161 (7)	0.01740 (13)	
O1	0.36843 (6)	0.5071 (4)	0.19698 (15)	0.0251 (4)	
C1	0.59800 (8)	0.0731 (5)	0.4638 (2)	0.0201 (4)	
H1	0.6276	0.0445	0.5161	0.024*	
C2	0.59738 (8)	−0.0357 (5)	0.3506 (2)	0.0201 (4)	
H2	0.6268	−0.1480	0.3143	0.024*	
C3	0.54817 (8)	0.0355 (5)	0.2914 (2)	0.0173 (4)	
H3	0.5412	−0.0249	0.2117	0.021*	
C4	0.51135 (8)	0.2025 (5)	0.36246 (19)	0.0153 (4)	
C5	0.45890 (8)	0.3086 (5)	0.32082 (19)	0.0152 (4)	
H5	0.4519	0.2571	0.2403	0.018*	
C6	0.41829 (8)	0.4681 (4)	0.37662 (18)	0.0149 (4)	
C7	0.36964 (8)	0.5503 (5)	0.30417 (19)	0.0157 (4)	
C8	0.32189 (8)	0.6897 (4)	0.36676 (19)	0.0149 (4)	
C9	0.27955 (8)	0.8271 (5)	0.3005 (2)	0.0170 (4)	
H9	0.2824	0.8382	0.2168	0.020*	
C10	0.23379 (8)	0.9464 (5)	0.35577 (19)	0.0177 (4)	
H10	0.2053	1.0389	0.3103	0.021*	
C11	0.22952 (8)	0.9309 (5)	0.47876 (19)	0.0179 (4)	
H11	0.1979	1.0100	0.5171	0.022*	
C12	0.27162 (9)	0.7996 (5)	0.5445 (2)	0.0176 (4)	
H12	0.2688	0.7920	0.6282	0.021*	
C13	0.31817 (7)	0.6783 (4)	0.4900 (2)	0.0140 (4)	
C14	0.36339 (8)	0.5298 (5)	0.56230 (18)	0.0149 (4)	
H14A	0.3570	0.2872	0.5728	0.018*	
H14B	0.3637	0.6348	0.6415	0.018*	
C15	0.41813 (7)	0.5824 (4)	0.5033 (2)	0.0150 (4)	
H15A	0.4276	0.8228	0.5064	0.018*	
H15B	0.4460	0.4573	0.5477	0.018*	

### Atomic displacement parameters (Å^2^)

**Table:**

	*U*^11^	*U*^22^	*U*^33^	*U*^12^	*U*^13^	*U*^23^
S1	0.0148 (2)	0.0213 (2)	0.0160 (2)	0.00044 (16)	−0.0005 (2)	−0.0013 (2)
O1	0.0217 (9)	0.0398 (9)	0.0137 (8)	0.0081 (6)	−0.0021 (6)	−0.0022 (7)
C1	0.0132 (9)	0.0204 (9)	0.0267 (12)	−0.0002 (7)	−0.0007 (8)	0.0033 (8)
C2	0.0145 (10)	0.0212 (10)	0.0247 (12)	0.0020 (7)	0.0048 (9)	0.0030 (9)
C3	0.0195 (10)	0.0168 (9)	0.0156 (10)	−0.0027 (7)	−0.0009 (8)	0.0041 (8)
C4	0.0174 (10)	0.0145 (8)	0.0139 (10)	−0.0021 (7)	−0.0012 (8)	0.0017 (8)
C5	0.0164 (10)	0.0178 (9)	0.0113 (10)	−0.0016 (7)	−0.0012 (7)	0.0011 (8)
C6	0.0162 (9)	0.0147 (9)	0.0137 (10)	−0.0011 (7)	−0.0007 (8)	0.0028 (8)
C7	0.0156 (10)	0.0168 (9)	0.0147 (10)	0.0009 (7)	−0.0004 (8)	0.0008 (8)
C8	0.0143 (9)	0.0138 (8)	0.0167 (10)	−0.0021 (7)	−0.0014 (8)	0.0012 (8)
C9	0.0182 (10)	0.0175 (9)	0.0153 (10)	−0.0021 (8)	−0.0019 (8)	0.0016 (8)
C10	0.0130 (9)	0.0177 (9)	0.0226 (12)	−0.0019 (7)	−0.0046 (8)	0.0028 (8)
C11	0.0150 (9)	0.0172 (9)	0.0216 (12)	−0.0008 (7)	0.0035 (8)	0.0002 (7)
C12	0.0173 (10)	0.0186 (10)	0.0169 (10)	−0.0033 (7)	0.0032 (8)	0.0012 (8)
C13	0.0155 (9)	0.0119 (8)	0.0147 (10)	−0.0029 (6)	−0.0001 (8)	0.0007 (8)
C14	0.0166 (9)	0.0152 (9)	0.0129 (10)	−0.0012 (7)	0.0010 (8)	0.0001 (8)
C15	0.0138 (8)	0.0187 (8)	0.0125 (9)	0.0005 (7)	−0.0006 (9)	−0.0001 (9)

### Geometric parameters (Å, º)

**Table:**

S1—C1	1.714 (2)	C8—C9	1.403 (3)
S1—C4	1.737 (2)	C9—C10	1.381 (3)
O1—C7	1.226 (3)	C9—H9	0.9500
C1—C2	1.353 (3)	C10—C11	1.397 (3)
C1—H1	0.9500	C10—H10	0.9500
C2—C3	1.421 (3)	C11—C12	1.385 (3)
C2—H2	0.9500	C11—H11	0.9500
C3—C4	1.388 (3)	C12—C13	1.396 (3)
C3—H3	0.9500	C12—H12	0.9500
C4—C5	1.447 (3)	C13—C14	1.510 (3)
C5—C6	1.349 (3)	C14—C15	1.527 (3)
C5—H5	0.9500	C14—H14A	0.9900
C6—C7	1.495 (3)	C14—H14B	0.9900
C6—C15	1.504 (3)	C15—H15A	0.9900
C7—C8	1.488 (3)	C15—H15B	0.9900
C8—C13	1.399 (3)		
			
C1—S1—C4	92.35 (10)	C10—C9—H9	119.7
C2—C1—S1	111.91 (16)	C8—C9—H9	119.7
C2—C1—H1	124.0	C9—C10—C11	119.89 (19)
S1—C1—H1	124.0	C9—C10—H10	120.1
C1—C2—C3	113.1 (2)	C11—C10—H10	120.1
C1—C2—H2	123.5	C12—C11—C10	119.65 (19)
C3—C2—H2	123.5	C12—C11—H11	120.2
C4—C3—C2	112.9 (2)	C10—C11—H11	120.2
C4—C3—H3	123.6	C11—C12—C13	121.2 (2)
C2—C3—H3	123.6	C11—C12—H12	119.4
C3—C4—C5	122.9 (2)	C13—C12—H12	119.4
C3—C4—S1	109.82 (15)	C12—C13—C8	118.9 (2)
C5—C4—S1	127.21 (16)	C12—C13—C14	120.8 (2)
C6—C5—C4	131.1 (2)	C8—C13—C14	120.26 (17)
C6—C5—H5	114.5	C13—C14—C15	111.67 (17)
C4—C5—H5	114.5	C13—C14—H14A	109.3
C5—C6—C7	116.72 (18)	C15—C14—H14A	109.3
C5—C6—C15	126.27 (18)	C13—C14—H14B	109.3
C7—C6—C15	116.97 (16)	C15—C14—H14B	109.3
O1—C7—C8	120.28 (18)	H14A—C14—H14B	107.9
O1—C7—C6	122.11 (18)	C6—C15—C14	112.16 (16)
C8—C7—C6	117.60 (18)	C6—C15—H15A	109.2
C13—C8—C9	119.78 (19)	C14—C15—H15A	109.2
C13—C8—C7	121.00 (18)	C6—C15—H15B	109.2
C9—C8—C7	119.20 (19)	C14—C15—H15B	109.2
C10—C9—C8	120.5 (2)	H15A—C15—H15B	107.9
			
C4—S1—C1—C2	0.54 (16)	C6—C7—C8—C9	168.28 (17)
S1—C1—C2—C3	−0.5 (2)	C13—C8—C9—C10	−1.1 (3)
C1—C2—C3—C4	0.2 (3)	C7—C8—C9—C10	177.44 (17)
C2—C3—C4—C5	178.49 (17)	C8—C9—C10—C11	0.1 (3)
C2—C3—C4—S1	0.2 (2)	C9—C10—C11—C12	0.8 (3)
C1—S1—C4—C3	−0.40 (15)	C10—C11—C12—C13	−0.8 (3)
C1—S1—C4—C5	−178.62 (18)	C11—C12—C13—C8	−0.2 (3)
C3—C4—C5—C6	179.2 (2)	C11—C12—C13—C14	−178.85 (17)
S1—C4—C5—C6	−2.8 (3)	C9—C8—C13—C12	1.1 (3)
C4—C5—C6—C7	177.95 (19)	C7—C8—C13—C12	−177.36 (16)
C4—C5—C6—C15	0.4 (3)	C9—C8—C13—C14	179.76 (16)
C5—C6—C7—O1	−7.2 (3)	C7—C8—C13—C14	1.3 (3)
C15—C6—C7—O1	170.53 (18)	C12—C13—C14—C15	−150.02 (17)
C5—C6—C7—C8	172.98 (17)	C8—C13—C14—C15	31.4 (2)
C15—C6—C7—C8	−9.3 (2)	C5—C6—C15—C14	−140.98 (19)
O1—C7—C8—C13	166.95 (18)	C7—C6—C15—C14	41.5 (2)
C6—C7—C8—C13	−13.2 (3)	C13—C14—C15—C6	−51.7 (2)
O1—C7—C8—C9	−11.5 (3)		
